# Analysis on the spatiotemporal pattern and driving force of the spatial deviation index of grain and economy in counties in China

**DOI:** 10.1371/journal.pone.0306970

**Published:** 2024-12-19

**Authors:** Jia Chen, Kuan Zhang

**Affiliations:** 1 Southwest Jiaotong University Hope College, Chengdu, Sichuan, China; 2 Sichuan Agricultural University, Ya’an, Sichuan, China; Gebze Teknik Universitesi, TÜRKIYE

## Abstract

Studying the spatial relationship and driving forces between grain production and economic development in China can assist in the coordinated development of economic growth and grain production in both China and other developing countries. Based on panel data from 2000 to 2019 covering 2018 county-level units in China, this study comprehensively investigated the spatial distribution, spatial differences, dynamic evolution of distribution, and driving factors of China’s county-level spatial deviation index of grain and economy (SDIGE) using methods such as the standard deviation ellipse method, the three-stage nested decomposition of Theil index, kernel density estimation, and geographically weighted regression (GWR) model. The results show that (1) from 2000 to 2019, China’s SDIGE showed a development trend of "up—down—up," and the highest SDIGE was in the northeast region, the lowest in the east region, and the spatial pattern of "high in the northeast—low in the east coast" was increasingly prominent. (2) In terms of spatial difference, the overall difference of SDIGE in China from 2000 to 2019 showed a rising trend of development; The average contribution rate of the regional difference to the overall difference was the lowest, maintained at about 17.82%; The average contribution rate of intra city and inter-county differences to the overall difference is the highest, which is about 34.20%. (3) In terms of the driving force, the level of economic development hurts SDIGE, while population density, industrial structure, fiscal decentralisation, and terrain fluctuation have a positive and negative impact on SDIGE. To alleviate the imbalance between China’s economic development and grain production, it is necessary to implement differentiated policy measures tailored to the specific characteristics of different regions to assist agricultural producers and enhance the stability of grain production.

## 1. Introduction

Food is the foundation of human survival and economic and social development. Since the Food Security Committee of the Food and Agriculture Organization of the United Nations adopted the concept of "food security" in 1983, more and more countries have begun to actively participate in food security governance, and have taken the improvement of food security as the primary task of promoting economic development and stabilising society. However, since 2014, the global food security situation has continued to deteriorate, with a growing number of people suffering from hunger [1]. According to the "2022 Global Food Crisis Report," in 2021, approximately 193 million people in 53 countries or regions experienced a food security crisis, an increase of nearly 40 million people compared to 2020, reaching the highest level since 2016 [2, 3]. The food crisis is posing challenges to the sustainable economic and social development of countries or regions. For example, Seal, Checchi [4] found that the worsening of the food crisis increased the risk of excessive deaths from infectious diseases in Somalia; Carril, Paniagua [5] and Sadiddin, Cattaneo [6] found that food crises compel people to engage in international migration; Meanwhile, Meriläinen, Mitrunen [7] discovered through historical data collected from Finland that famine increases income and land distribution inequality, thereby leading to large-scale conflicts and rebellions. Of course, the challenge of food security goes far beyond those highlighted in the above-mentioned studies.

Grain is a fundamental necessity upon which humanity relies for survival, providing the material foundation for economic development. As the world’s most populous country and the second-largest economy [8], China has taken many policy measures to accelerate regional economic development and improve food security. However, there still exists a certain degree of conflict between regional economic development and grain production, which is detrimental to both the stable development of the economy and the enhancement of food security capabilities [9–11]. In 2006, China fully abolished agricultural taxes, leading to a situation where grain production no longer contributes to local government tax revenue. Moreover, many grain subsidy policies require complementary support from local governments, causing grain-producing counties to fall into the dilemma of " the more food, the poorer the county" [12, 13]. According to statistics, 105 out of the 800 major grain-producing counties in China were state-level poverty-stricken counties in 2017, with about 36 million poor people [14]. Counties, as the basic units of national economic development, play a fundamental role in achieving regional coordinated development. In this context, conducting a comprehensive examination of the spatiotemporal patterns of the spatial deviation index of grain and economy (SDIGE) at the county level, and analyzing its driving factors, is of significant reference value for formulating policies that promote the coordinated development of regional economic growth and grain production.

From existing literature, it is evident that scholars have extensively researched the relationship between economic development and grain production in China. In terms of the spatiotemporal pattern of grain production, the centre of grain production is shifting towards the north, and a spatial pattern of "contraction in the east, expansion in the central regions" is observed [15–17]. This is attributed to the continuous reduction of arable land in regions with better economic development, as well as changes in the structure of arable land, leading to changes in the spatial pattern of grain production in China [18, 19]. In terms of the influencing factors of food production, scholars discussed the impact of climate change, production transformation, technological progress, and cultural differences on food production [20–23]. For example, Xu, Zhang [24] found that the impact of drought on the grain production system showed the basic law of "marginal loss decreasing"; Tang, Lu [25] found that the spatial spillover effect of farmland utilisation transformation on food production is heterogeneous due to terrain constraints, that is, in plain areas, it mainly depends on machinery input, farmland quantity and farmland infrastructure input, while it depends on labour input in hilly areas. As far as the spatial relationship between economic development and food production is concerned, Hou, Deng [26] and Xie, Ding [14] both believe that food agglomeration in northeast China is stronger than economic agglomeration, while food agglomeration in east China is weaker than economic agglomeration. However, some scholars found that the focus of China’s grain production was moving to the northwest [27], necessitating the need for further research on the spatial relationship between economic development and grain production.

Through reviewing relevant literature, it is found that research on the spatial relationship between economic development and grain production primarily focuses on the provincial level. There is scarce literature that examines the spatiotemporal patterns and driving forces of China’s Spatial Deviation Index of Grain and Economy (SDIGE) at the county level. For China, a country with a population of 1.4 billion, the government should not occupy the space for food production, while pursuing rapid economic development, that is, to prevent the "economic expulsion of grain," but should comprehensively consider the coordinated development of economy and food in the region. Therefore, the study of the spatial deviation index with dual effects of economic development and food production can, to a certain extent, clarify the spatial relationship between China’s economic development and food production and thus provide a reference for ensuring food security and sustainable economic development, which is more of practical significance and theoretical value.

In contrast to previous studies, and based on the panel data of 2018 county units in China from 2000 to 2019, this paper comprehensively investigates the spatial distribution, spatial differences, dynamic evolution of distribution, and driving factors of the spatial deviation between China’s county economy and food by using methods such as three-stage nested decomposition of the Thiel index and geographically weighted regression (GWR) model. The marginal contribution of this paper is mainly reflected in the following four aspects: (1) On the sample data, this paper uses county unit panel data for the first time to comprehensively investigate the spatial and temporal pattern and evolution trend of SDIGE in China; (2) On the spatial scale, this paper uses the three-stage nested decomposition method of Thiel index to expand the decomposition level of SDIGE spatial difference sources, which can reveal the internal structural characteristics of China’s SDIGE spatial differences in more detail; (3) In terms of indicator measurement, this paper measures the SDIGE at the county level in China, which helps to clarify the source of spatial differences in SDIGE and the geographic scale of China’s SDIGE. The proposed method can provide effective measurement and can be used in other similar situations; (4) In terms of driving force analysis, the GWR model adopted in this paper can solve the estimation error caused by spatial correlation, which is conducive to revealing the spatial relationship between SDIGE and its driving forces, and can put forward more accurate policy recommendations. The rest of this paper is arranged as follows: The second section describes the methods and data used in this paper in detail; The third section analyses the temporal and spatial pattern of China’s county SDIGE; The fourth section analyses the driving force of China’s county SDIGE; The fifth section is the discussion of this paper; The sixth section is the conclusions and policy implications of this paper.

## 2. Research methods and data

### 2.1. Thiel index

Theil index was first proposed by Theil [28]. It is an important indicator to measure the degree of differences between individuals, and can intuitively analyse regional differences and sources. However, the traditional Thiel index can only be carried out in one order and is limited to two orders. To solve the above problems, Akita [29] proposed a second-order nested decomposition method based on the traditional Thiel index. As the county unit is the basic spatial unit in this paper, we propose the use of the third-order nested decomposition method of the Thiel index to decompose the four levels of zone, province, city and county, and reveal and compare the structural characteristics of the spatial differences of China’s SDIGE at each level and their contribution to the overall differences. In this paper, *I*, *j*, *k*, *l* are designated as zones, provinces, cities, and counties, respectively, including the eastern, central, western, and northeastern regions of China. If the county-level data is taken as the research sample and only the decomposition within and between zones is considered, the traditional decomposition formula of the Thiel index is as follows:

$$Theil\;=\sum_{i}\sum_{l}\frac{{SDIGE}_{il}}{SDIGE}log\frac{{sdige}_{il}}{sdige}=\sum_{i}\sum_{l}\frac{{SDIGE}_{il}}{SDIGE}log\left(\frac{{sdige}_{i}}{sdige}\times \frac{{sdige}_{il}}{{sdige}_{i}}\right)=\sum_{i}\sum_{l}\frac{{SDIGE}_{il}}{SDIGE}log\frac{{SDIGE}_{il}}{SDIGE}log\frac{{sdige}_{i}}{sdige}+\sum_{i}\sum_{l}\frac{{SDIGE}_{il}}{SDIGE}log\frac{{SDIGE}_{il}}{SDIGE}log\frac{{sdige}_{il}}{{sdige}_{i}}=\sum_{i}\frac{{SDIGE}_{i}}{SDIGE}log\frac{{sdige}_{i}}{sdige}+\sum_{i}\sum_{l}\frac{{SDIGE}_{il}}{SDIGE}log\frac{{sdige}_{il}}{{sdige}_{i}}$$ (1)

Where *Theil* is the Theil index and *sdige* is the average value of *SDIGE* in each zone. $\sum_{i}\frac{{SDIGE}_{i}}{SDIGE}log\frac{{sdige}_{i}}{sdige}$ is the expression of difference between zones, and $\sum_{i}\sum_{l}\frac{{SDIGE}_{il}}{SDIGE}log\frac{{sdige}_{il}}{{sdige}_{i}}$ is the expression of difference between counties within the zone. Based on the county difference expression within the zone, the index at the municipal level can be transformed into Formula (2).


$$\sum_{i}\sum_{l}\frac{{SDIGE}_{il}}{SDIGE}log\frac{{sdige}_{il}}{{sdige}_{i}}=\sum_{i}\frac{{SDIGE}_{i}}{SDIGE}\left(\sum_{k}\sum_{l}\frac{{SDIGE}_{ikl}}{{SDIGE}_{i}}log\frac{{sdige}_{ikl}}{{sdige}_{i}}\right)$$ (2)

By analogy, expression $\sum_{k}\sum_{l}\frac{{SDIGE}_{ikl}}{{SDIGE}_{i}}log\frac{{sdige}_{ikl}}{{sdige}_{i}}$ can also be regarded as the Thiel index of each zone level, and can be further decomposed into inter city differences and intra city differences using the Thiel index decomposition method. The formula is shown in Eq (3).


$$\sum_{k}\sum_{l}\frac{{SDIGE}_{ikl}}{{SDIGE}_{i}}log\frac{{sdige}_{ikl}}{{sdige}_{i}}=\sum_{k}\sum_{l}\frac{{SDIGE}_{ikl}}{{SDIGE}_{i}}log\left(\frac{{sdige}_{ik}}{{sdige}_{i}}\times \frac{{sdige}_{ikl}}{{sdige}_{ik}}\right)=\sum_{k}\sum_{l}\frac{{SDIGE}_{ikl}}{{SDIGE}_{i}}log\frac{{sdige}_{ik}}{{sdige}_{i}}+\sum_{k}\sum_{l}\frac{{SDIGE}_{ikl}}{{SDIGE}_{i}}log\frac{{sdige}_{ikl}}{{sdige}_{ik}}=\sum_{k}\frac{{SDIGE}_{ik}}{{SDIGE}_{i}}log\frac{{sdige}_{ik}}{{sdige}_{i}}+\sum_{k}\sum_{l}\frac{{SDIGE}_{ikl}}{{SDIGE}_{i}}log\frac{{sdige}_{ikl}}{{sdige}_{ik}}$$ (3)

In the same way, we further decompose the provincial level indicators based on the intercity difference expression, and finally decompose the Theil index into formula (4).


$$T=\sum_{i}\frac{{SDIGE}_{i}}{SDIGE}log\frac{{sdige}_{i}}{sdige}+\sum_{i}\sum_{j}\frac{{SDIGE}_{ij}}{SDIGE}log\frac{{sdige}_{ij}}{{sdige}_{i}}+\sum_{i}\sum_{j}\sum_{k}\frac{{SDIGE}_{ijk}}{SDIGE}log\frac{{sdige}_{ijk}}{{sdige}_{ij}}+\sum_{i}\sum_{j}\sum_{k}\sum_{l}\frac{{SDIGE}_{ijkl}}{SDIGE}log\frac{{sdige}_{ijkl}}{{sdige}_{ij}k}$$ (4)

In Formula (4), the four parts of expression are, in turn, the difference between zones, the difference between provinces within the zone, the difference between cities within the province, and the difference between counties within the city. The essence of decomposition is to further decompose the intra zone differences in the traditional Theil index into inter provincial differences, intercity differences within provinces, and inter county differences within cities.

### 2.2. Spatial correlation test

It is necessary to check the space-time transition characteristics and spatial convergence of *SDIGE* before using the *GWR* model, i.e., to conduct spatial autocorrelation test. Therefore, this paper uses the global Moran’s I index to test spatial autocorrelation [30]. This paper also uses ArcGIS10.2 software and spatial inverse distance weight matrix to analyse the spatial correlation of China’s county *SDIGE*. The global Moran’s I index is calculated as follows.


$$I=\frac{n}{\sum_{i=1}^{n}\sum_{j=1}^{n}w_{ij}}\times \frac{\sum_{i=1}^{n}\sum_{j=1}^{n}w_{ij}\left({SDIGE}_{i}-\bar{SDIGE}\right)\left({SDIGE}_{j}-\bar{SDIGE}\right)}{\sum_{i=1}^{n}{\left({SDIGE}_{i}-SDIGE\right)}^{2}}$$ (5)

In Eq (5), *I* represents the global Moran index, *n* represents the number of observations, and *w*_*ij*_ represents the spatial inverse distance weight matrix of positions *i* and *j*; *SDIGE*_*i*_ and *SDIGE*_*j*_ are the observed values of area *i* and area *j*, respectively, and $\bar{SDIGE}$ is the average of *SDIGE* observations.

### 2.3. Geographically weighted regression model

The first law of geography states: "Everything is related to everything else, but near things are more related than distant things." Regional economic and social development is not independent of each other but is influenced by neighbouring regions. Therefore, there exists spatial correlation and spatial heterogeneity among individuals. The geographically weighted regression (*GWR*) model was initially proposed by Brunsdon, Fotheringham [31] based on the theory of local smoothing. In practical research, it is often found that regression coefficients vary across different regions, indicating that the coefficients change with geographical location. Compared to ordinary linear regression (*OLR*) models, the *GWR* model can effectively address this issue. Additionally, *GWR* can mitigate estimation errors caused by spatial correlation and explore spatial non-stationarity of variables from a local perspective. Therefore, this study employs spatial econometric analysis of the *SDIGE* using the spatial relationship modelling tool in ArcGIS 10.2 software. The calculation formula of *GWR* is as follows.


$$SDIGE_{i}=\beta_{0}\left(x_{i},\;y_{i}\right)+\sum_{j=1}^{k}\beta_{j}\left(x_{i},\;y_{i}\right)W_{ij}+\varepsilon_{i}$$ (6)

In Formula (6), *SDIGE*_*i*_ represents the observation value of the *i*-th sample; (*x*_*i*_, *y*_*i*_) is the spatial coordinate of the ith sample; *β*_*j*_(*x*_*i*_, *y*_*i*_) is the regression coefficient of the influencing factor *j* in the ith sample; *W*_*ij*_ is the *j*-th influencing factor of the ith unit *SDIGE*; and *ε*_*i*_ is a random error.

### 2.4. SDIGE

To analyse the spatial interrelationship between economic development and food production, this paper draws on Yang and He [32] and Xie, Ding (14)to measure the *SDIGE* index using two indicators: the geographical concentration of grain and the geographical concentration of the economy. *SDIGE* reflects the degree of deviation between economic development and grain production and shows the development balance of the study area and its adjacent areas on a spatial scale. The specific calculation formula is as follows.


$$SDIGE_{i}=\frac{{GY}_{i}/\sum_{i=1}^{n}GY_{i}}{G{DP}_{i}/\sum_{i=1}^{n}GDP_{i}}$$ (7)

In formula (7), *SDIGE*_*i*_ represents the spatial deviation of economy and grain in region *i*; ${GY}_{i}/\sum_{i=1}^{n}GY_{i}$ is the geographical concentration of grain, and *GY*_*i*_ is the grain output in region *i*; $G{DP}_{i}/\sum_{i=1}^{n}GDP_{i}$ is the economic geographical concentration, and *GDP*_*i*_ is the *GDP* of region *i*.

### 2.5. Variable selection

According to the regional comparative advantage theory, geographical conditions are the congenital factors that affect industrial layout, and industrial layout is an important factor affecting economic development and grain production [33]. Therefore, this paper conducts the initial selection of the driving factor indicator system from the two dimensions of socio-economic factors and geographical conditions before using the GWR model to investigate the driving factors of SDIGE. In terms of social economy, this paper selects eight independent variables of SDIGE, including economic development level, agricultural mechanisation level, population density, industrial structure, energy consumption intensity, environmental regulation, fiscal decentralisation, and financial development level. In terms of geographical conditions, this paper selects three variables, namely topographic relief, provincial border counties, and elevation. These variables reflect the socio-economic and geographical conditions of each county and are highly related to the food production and economic development of each county.

### 2.6. Data

This paper selects 2018 county units from 31 provinces (autonomous regions, municipalities) in China (excluding Tibet, Hong Kong, Macao and Taiwan) from 2000 to 2019 as research samples. The selected 31 provinces (autonomous regions, municipalities) are divided into the eastern, central, western, and northeast regions, according to China’s regional division method of the National Bureau of Statistics in 2011. Among the research samples, there are 498 county units in the east, 492 in the middle, 849 in the west, and 179 in the northeast.

Based on the feasibility and integrity of data acquisition, this paper obtains research data through multiple channels. Among them, grain production, economic development level, level of agricultural mechanisation, population density, industrial structure, fiscal decentralisation and level of financial development are taken from the EPS database (https://www.epsnet.com.cn/), the *China County Statistical Yearbook*, and the country’s *National Economic and Social Development Statistical Bulletin*. The missing data of individual years are supplemented by interpolation. The energy consumption intensity data comes from the research of Chen, Gao [34]. The environmental regulation data comes from the Social Economic Data and Application Center of Columbia University (https://sedac.ciesin.columbia.edu/). The topographic relief data is derived from the research of Zhongqian [35]. The altitude data comes from ASTER Global Digital Elevation Model V003 (https://search.earthdata.nasa.gov/) Digital Elevation Model (DEM), with a global resolution of 30 meters. The provincial boundary county data is identified and obtained using ArcGIS10.2 software based on the administrative division map of each province (autonomous regions, municipalities). To reduce heteroscedasticity and eliminate the impact of variable dimensions, this paper standardised all independent variables with natural logarithms, except for provincial border counties. Table 1 shows the detailed introduction and descriptive statistical analysis of variables.

**Table 1:** Variable introduction and descriptive statistics.

Variables	Meaning and assignment	Mean	SD
SDIGE	Spatial deviation index of grain and economy	1.520	1.515
Economic development level	County gross regional product per capita (10000 yuan)	2.400	2.950
Agricultural mechanisation level	Ratio of total power of agricultural machinery to rural employees (MW/ 1000 persons)	2.224	5.510
Population density	County population/county administrative area (persons/ km2)	330.158	412.058
Industry Structure	Value added of secondary industry/ value added of tertiary industry	0.409	0.157
Intensity of energy consumption	Power consumption/ GDP (KWh/ yuan)	0.284	1.126
Environmental regulation	The reciprocal of PM2.5	0.031	0.042
Fiscal decentralisation	Per capita fiscal expenditure of local government/ per capita fiscal expenditure of central government	3.026	2.944
Financial development level	Total loans from financial institutions/ GDP	0.608	0.456
Topographic relief	Topographic relief of the county	1.052	1.234
Provincial border counties	Whether the county is located at the provincial border: Yes = 1, No = 0	0.389	0.488
Elevation	Average elevation of the county (m)	594.790	769.376

## 3. The spatiotemporal pattern of SDIGE in Chinese counties

### 3.1. Spatial distribution characteristics of SDIGE

**Fig 1:**
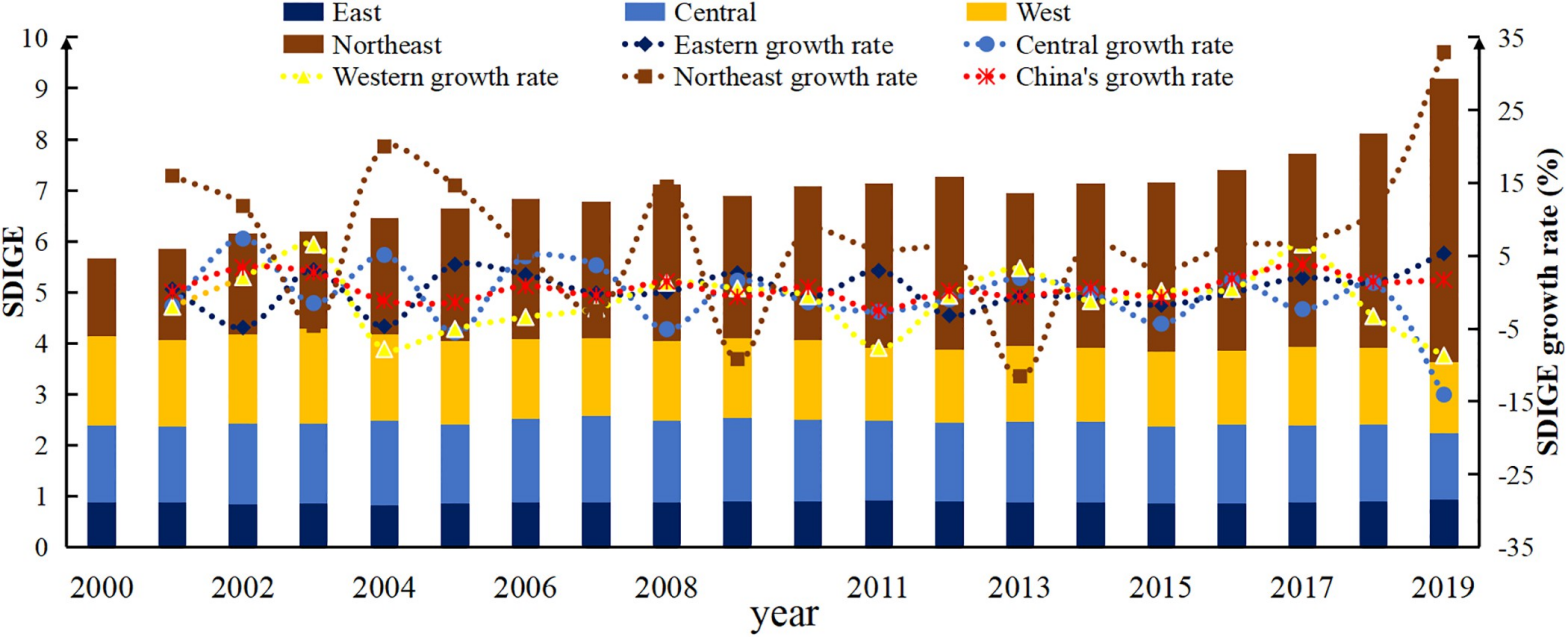
This figure illustrates the evolution trend of SDIGE from 2000 to 2019 in China, as well as in its eastern, central, western, and northeastern regions. The SDIGE values are represented by stacked columns for each region, while the corresponding growth rates are depicted by dashed lines. The dark blue columns represent the SDIGE values for the eastern region, blue columns for the central region, yellow columns for the western region, and brown columns for the northeastern region. The red dashed line represents the SDIGE growth rate for China, the dark blue dashed line for the eastern region, the blue dashed line for the central region, the yellow dashed line for the western region, and the brown dashed line for the northeastern region. The SDIGE values are plotted on the left vertical axis, the growth rates are plotted on the right vertical axis, and the years are plotted on the horizontal axis.

As shown in Fig 1, the evolution trend of China’s SDIGE has mainly gone through three stages. In the first stage (2000–2003), China’s SDIGE showed a growing trend, with an average growth rate of 2.05%; In the second stage (2004–2015), China’s SDIGE showed a trend of fluctuation and decline, with an average growth rate of -0.30%, indicating a phenomenon of economic development occupying the space for grain production; In the third stage (2016–2019), China’s SDIGE showed a rising development trend, with an average growth rate of 2.20%, indicating that the phenomenon of "economic expulsion of grain" has eased. In terms of regional distribution, the average SDIGE index is highest in the northeastern region, while it is lowest in the eastern region. During the study period, SDIGE in Northeast China and East China showed an upward trend, rising from 1.534 to 5.577 and 0.877 to 0.935, respectively. However, the average SDIGE in East China was the lowest, at 0.876. SDIGE in the central and western regions showed a declining trend, from 1.509 to 1.305 and 1.743 to 1.380, respectively. From the perspective of growth rate, only the northeast and eastern regions have positive average growth rates of SDIGE, 7.50% and 0.37%, respectively. The average growth rate of SDIGE in the central and western regions is negative, -0.65% and -1.14%, respectively, indicating that economic development and food production in the western region are not coordinated.

**Fig 2:**
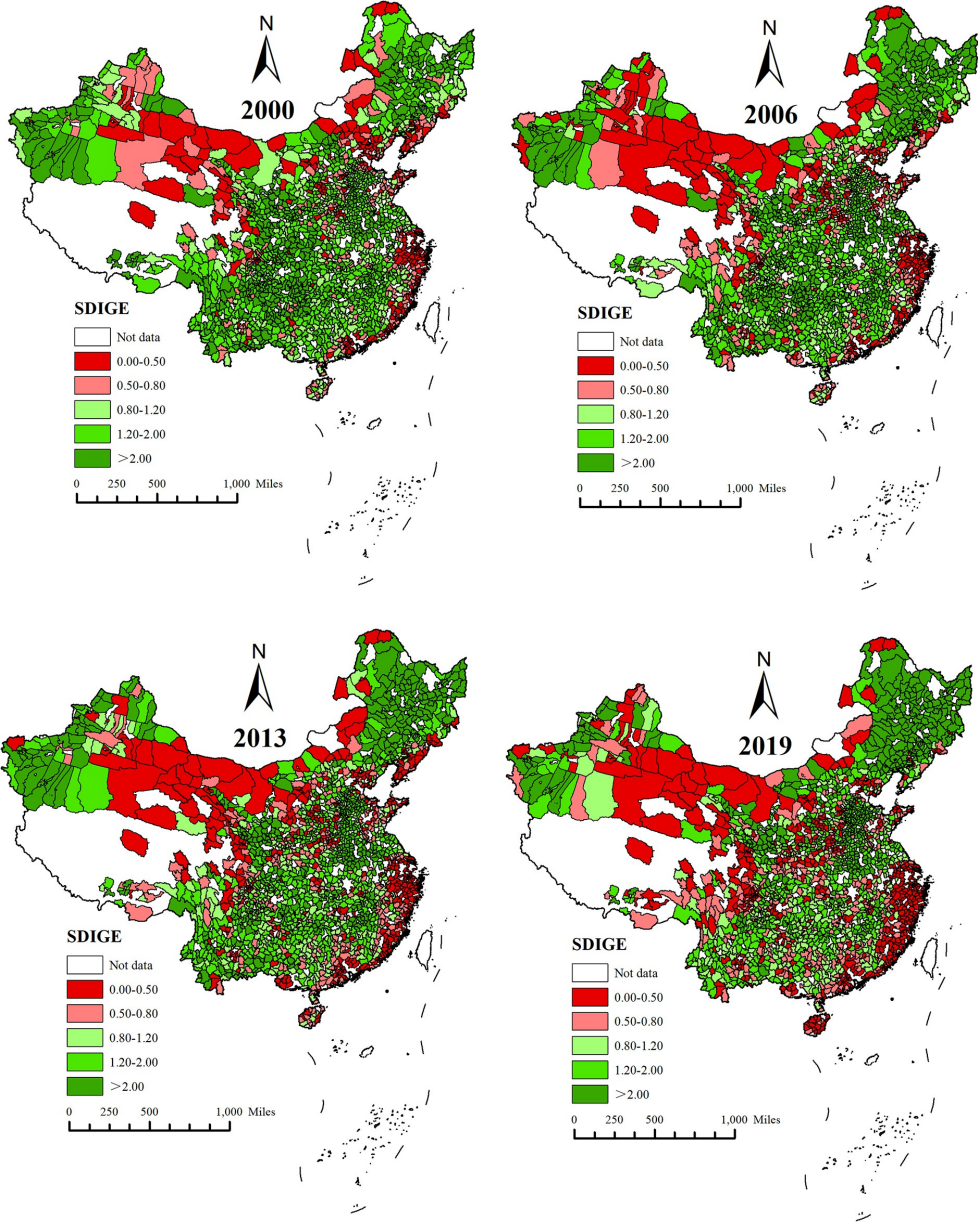
This figure, created using ArcGIS software, illustrates the spatial distribution characteristics of SDIGE in China for the years 2000, 2006, 2013, and 2019. In the figure, the white areas indicate missing data, the red areas represent SDIGE values between 0 and 0.5, the pink areas represent SDIGE values between 0.5 and 0.8, the light apple green areas represent SDIGE values between 0.8 and 1.2, the teal areas represent SDIGE values between 1.2 and 2.0, and the leaf green areas represent SDIGE values greater than 2.

Based on the analysis of SDIGE using ArcGIS10.2 software, this paper divides the SDIGE index into five conventional grades, as shown in Fig 2. In 2000, the SDIGE of most counties in China was higher than 0.80, while counties with lower SDIGE were mainly concentrated in the eastern coastal areas and some northwest areas. Compared with 2000, 2006, 2013 and 2019, the spatial distribution difference of SDIGE has significantly increased. The spatial pattern of "high in the northeast—low in the east coast" has become increasingly prominent. The spatial pattern of "high in the southwest—low in the northwest" has gradually evolved into "high in north China—low in the northwest." Specifically, the SDIGE of county units in Northeast China, especially in Gannan County and Raohe County in Heilongjiang Province, Changtu County in Liaoning Province, and Qian’an County in Jilin Province, has gradually increased. In 2019, the SDIGE exceeded 12. The second is the county units in the central and western regions, Shenchi County and Wuzhai County in Shanxi Province, Huining County and Tongwei County in Gansu Province. In 2019, SDIGE exceeded five. The SDIGE of county units in the eastern region is low, and more than 65% of the sample in 2019 had SDIGE lower than one.

### 3.2. The centre of gravity shift and SDE analysis of SDIGE

**Fig 3:**
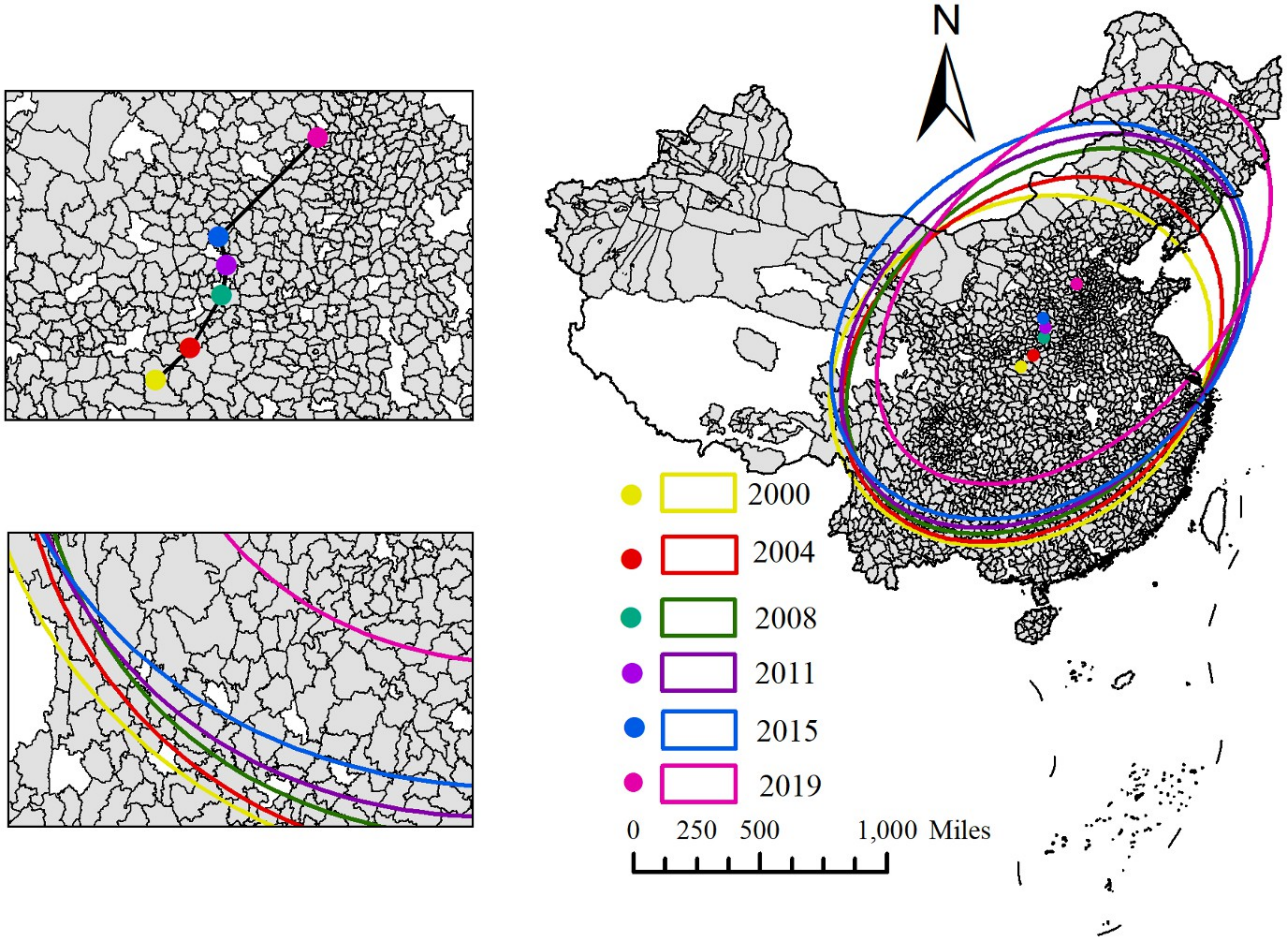
This figure, created using ArcGIS software, illustrates the migration paths of SDIGE in China. The yellow dots and circles represent the centroid and standard deviation ellipse of SDIGE in 2000, respectively. The red dots and circles represent the centroid and standard deviation ellipse of SDIGE in 2004, the green dots and circles for 2008, the purple dots and circles for 2011, the blue dots and circles for 2015, and the tan dots and circles for 2019.

This paper selects six characteristic time points in 2000, 2004, 2008, 2011, 2015, and 2019 to calculate the transfer trajectory and SDE-related attributes of China’s SDIGE gravity centre (see Fig 3). As shown in Fig 3, from the track and direction of gravity centre movement, the distribution centre of SDIGE in China moves from Shangzhou District to Lingbao City, Yuanqu County, Fushan County, Hongdong County and Luquan District along the northeast, with a moving distance of about 628.530 km. In general, the centre of gravity of China’s SDIGE distribution moves the largest distance to the northeast, indicating that the growth rate of county SDIGE in northeast China is far higher than the average level in China.

From 2000 to 2019, the SDE of China’s SDIGE was mainly located in the eastern, central, northeast and southwest regions of China, basically showing the spatial distribution pattern of "northeast-southwest." The ellipse area in 2019 is 8.92% larger than in 2000, indicating that SDIGE in China has a trend of spatial divergence. From the perspective of azimuth, the turning angle shows a gradually narrowing trend of development, and the range of change is large, indicating that the direction of SDIGE divergence in China is not stable. The length of the long semi-axis gradually expanded from 1281.091 km in 2000 to 1545.628 km in 2019, and the length of the short semi-axis gradually decreased from 1058.208km in 2000 to 955.378 km in 2019, indicating that the SDIGE of the county units in China is spatially divergent from northeast to southwest and concentrically concentrated from northwest to southeast.

### 3.3. Analysis of spatial evolution characteristics of SDIGE

**Fig 4:**
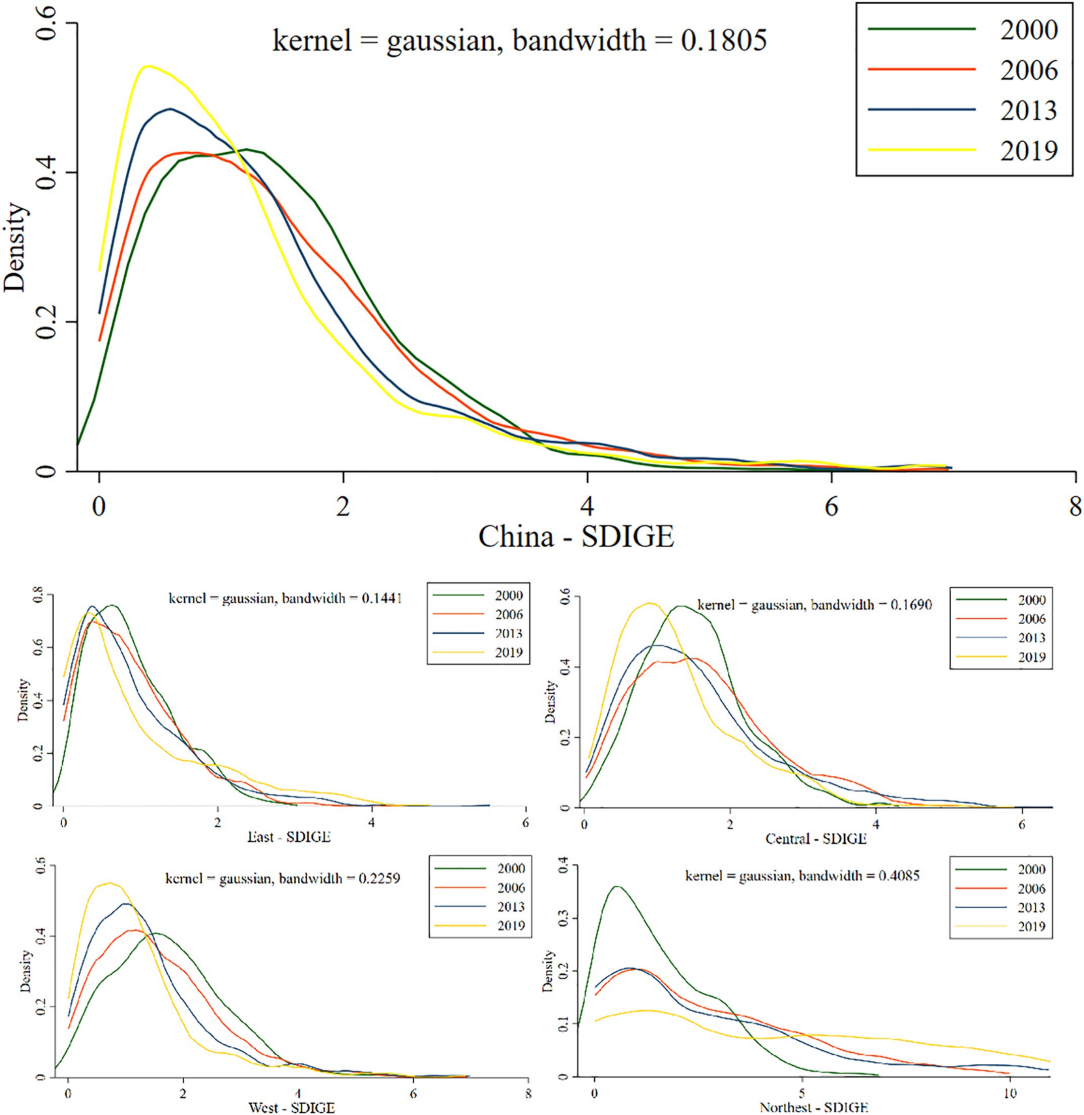
This figure, created using Stata software, illustrates the temporal evolution characteristics of SDIGE in China and its four major regions. The horizontal axis represents the range of SDIGE values, and the vertical axis represents the probability density at corresponding values. The kernel function is specified for kernel regression, and the bandwidth indicates the degree of smoothing in the estimation. The green line represents the density distribution of SDIGE in 2000, the red line represents 2006, the blue line represents 2013, and the yellow line represents 2019.

Using Stata 14 software, the nuclear density curves of SDIGE in China’s county units in 2000, 2006, 2013 and 2019 were drawn (see Fig 4). According to the location, shape, peak value, and extensibility of the nuclear density estimation curve, the dynamic evolution law of SDIGE time series was revealed.

#### 3.3.1. Global time series dynamic evolution characteristics

From a holistic perspective, the temporal evolution characteristics of the SDIGE across county-level units in China can be depicted using kernel density estimation. (1) From the change of the centre of gravity position of the curve, it moved to the left from 2000 to 2006 and to the right from 2006 to 2019, indicating that China’s SDIGE showed an evolutionary feature of first falling and then rising during the study period. (2) From the number of wave peaks in the curve, the degree of sub-peaks in 2000 is not obvious, and the sub-peaks in 2006–2019 disappear, indicating that the trend of SDIGE monopolarisation is relatively stable. (3) From the curve peak kurtosis, the curve kurtosis from 2000 to 2019 showed an obvious "downward upward" trend, indicating that the SDIGE difference between counties showed a trend of first expanding and then narrowing. (4) From the curve tail, the right side of the curve was more trailing than the left side in 2000–2019, and the right side showed a trend of lengthening and thinning, indicating that the SDIGE in high-value districts and counties increased, and the number of county units in high-value districts decreased. Thus, SDIGE in China at different stages has dynamic evolution characteristics of different development levels, regional differences, and polarization degrees.

#### 3.3.2. Dynamic evolution characteristics of local time series

From the perspective of the four major regions, the temporal evolution characteristics of SDIGE in different regions of China can be depicted. (1) From the change of the centre of gravity position of the curve, the nuclear density curve in the central and western regions gradually moved to the left from 2000 to 2019, indicating that SDIGE was in a declining evolution trend. However, the nuclear density curves in Northeast China and East China are gradually moving to the right, indicating that SDIGE is in a rising trend. (2) From the number of wave peaks in the curve, there was only one wave peak in the eastern, central, and western regions from 2000 to 2019, indicating that SDIGE was relatively stable in a unipolar state; The coexistence of one main peak and one secondary peak in Northeast China shows that SDIGE is polarised. (3) From the curve peak kurtosis, from 2000 to 2019, the curve kurtosis in the eastern region showed an obvious "downward—upward—downward" trend, the central region showed a "downward—upward" trend, the western region showed a rising trend, and the northeast region showed a declining trend. (4) From the curve tail, the right tail of the curve of the four major zones was larger than the left tail from 2000 to 2019, and the right tail showed a trend of lengthening and thinning, indicating that the SDIGE of the counties in high-value areas was improved, and the proportion of the number of county units in high-value areas was reduced. Therefore, the dynamic evolution process of SDIGE time series in different zones is different at the local scale as a result of the superposition and symbiosis of regional space-time characteristics.

### 3.4. Spatial differences in SDIGE and their decomposition

**Fig 5:**
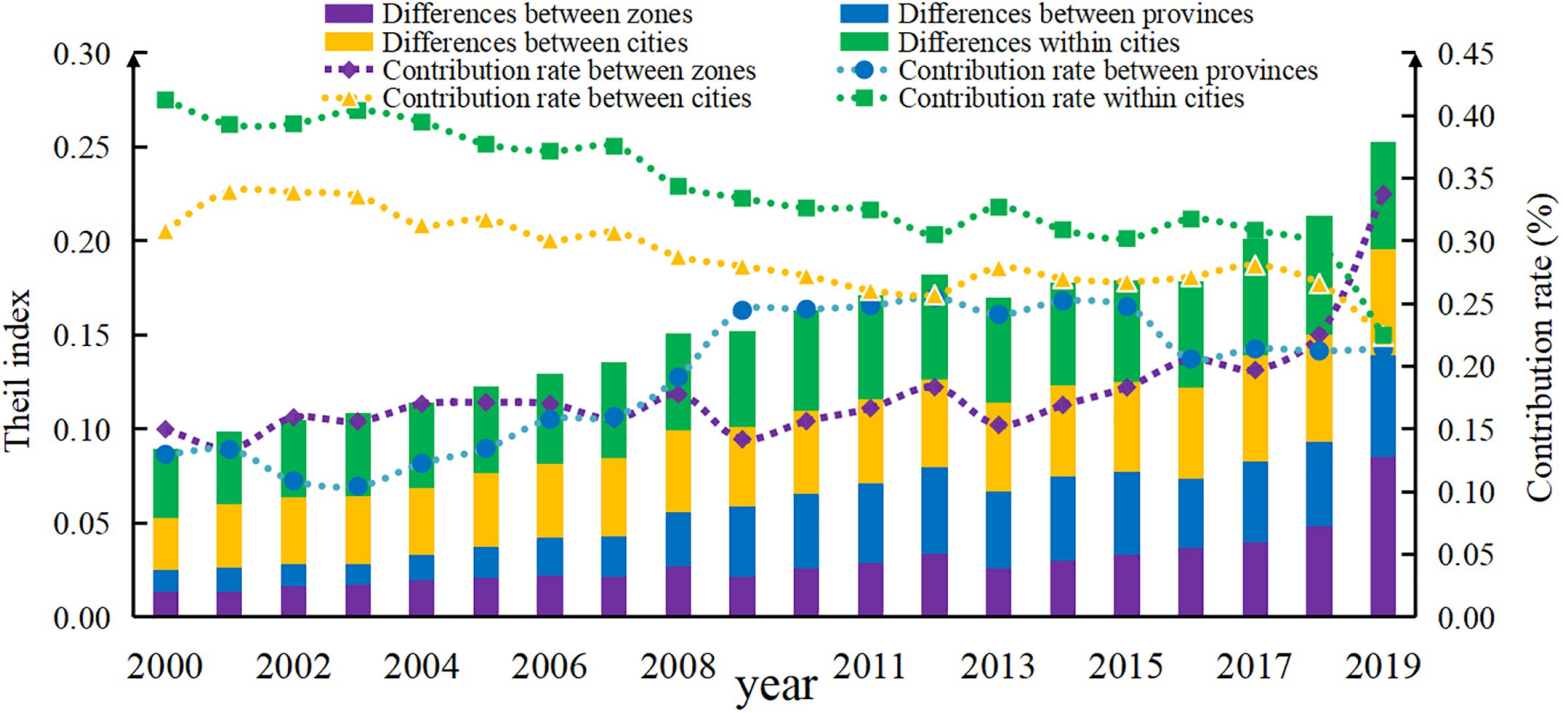
This figure shows the nested decomposition of China’s SDIGE Theil index into three stages from 2000 to 2019. The SDIGE Theil index is represented by stacked columns, while the corresponding growth rates are depicted by dashed lines. The purple columns represent the inter-regional differences in China’s SDIGE, the blue columns represent the inter-provincial differences within regions, the yellow columns represent the inter-municipal differences within provinces, and the green columns represent the inter-county differences within municipalities. The purple dashed line represents the contribution rate of inter-regional differences to the overall differences, the blue dashed line represents the contribution rate of inter-provincial differences within regions, the yellow dashed line represents the contribution rate of inter-municipal differences within provinces, and the green dashed line represents the contribution rate of inter-county differences within municipalities. The SDIGE Theil index is plotted on the left vertical axis, the contribution rates are plotted on the right vertical axis, and the years are plotted on the horizontal axis.

According to the three-stage nested decomposition formula of the Thiel index, this paper analyses the differences between zones, provinces, cities and counties in China’s SDIGE (see Fig 5). (1) During the sample survey, the overall difference of SDIGE in China showed a rising trend, from 0.089 in 2000 to 0.253 in 2019. (2) The regional difference of SDIGE in China shows a rising trend, rising from 0.013 in 2000 to 0.085 in 2019. Its contribution to the overall difference is also rising, but the average contribution to the overall difference, at about 17.82%, is the lowest. (3) The inter-provincial differences in China’s SDIGE region also show a rising trend, rising from 0.012 in 2000 to 0.054 in 2019. However, its contribution to the overall differences shows a trend of "decline—rise—gentle rise—decline". (4) The difference between cities in SDIGE province in China is on the rise, from 0.028 in 2000 to 0.057 in 2019, and its contribution to the overall difference is on the decline. (5) The intra-county difference of SDIGE in China shows a rising trend, from 0.037 in 2000 to 0.057 in 2011. Its contribution to the overall difference shows a declining trend, but it is still the largest source of the overall difference, about 34.20%.

## 4. Analysis of the driving forces of county SDIGE in China

### 4.1. Spatial Correlation Test of SDIGE

#### 4.1.1. Global spatial correlation of SDIGE

This paper uses ArcGIS10.2 software and spatial inverse distance weight matrix to calculate Moran’s I index of SDIGE in China (see Table 2). According to Table 2, Moran’s I index of SDIGE in China was positive from 2000 to 2019, showing a rising development trend; it passed the significance test at the level of 1%. Therefore, the distribution of SDIGE in China is spatially positive autocorrelation and spatially clustered rather than randomly distributed.

**Table 2:** Moran’s I Index of the SDIGE values of China’s counties.

Year	I	Z	P-value	Year	I	Z	P-value
2000	0.294	79.908	0.000	2010	0.425	116.132	0.000
2001	0.301	81.795	0.000	2011	0.442	120.886	0.000
2002	0.307	83.410	0.000	2012	0.466	127.320	0.000
2003	0.283	76.912	0.000	2013	0.427	116.463	0.000
2004	0.302	81.964	0.000	2014	0.448	122.155	0.000
2005	0.369	100.300	0.000	2015	0.468	127.583	0.000
2006	0.383	104.324	0.000	2016	0.453	123.300	0.000
2007	0.359	97.840	0.000	2017	0.450	122.566	0.000
2008	0.403	110.228	0.000	2018	0.482	131.475	0.000
2009	0.409	111.648	0.000	2019	0.614	167.313	0.000

#### 4.1.2. LISA agglomeration map of SDIGE

**Fig 6:**
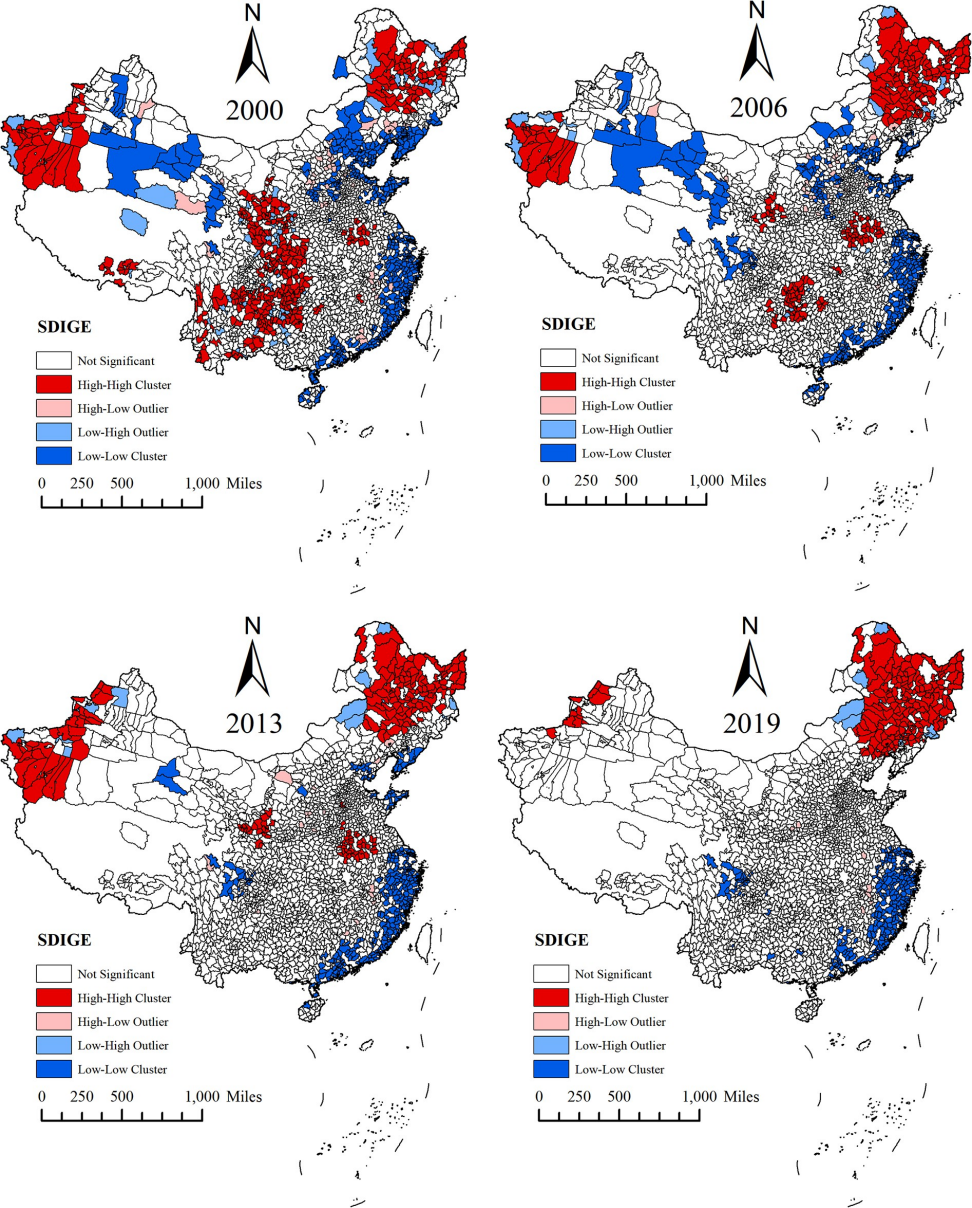
This figure, created using ArcGIS software, illustrates the LISA clustering maps of SDIGE in China for the years 2000, 2006, 2013, and 2019. In the figure, white areas indicate no significant differences, red areas represent high-value clusters, pink areas represent high-value outliers surrounded mainly by low values, light blue areas represent low-value outliers surrounded mainly by high values, and dark blue areas represent low-value clusters.

This paper further conducted a local spatial autocorrelation analysis on China’s SDIGE in 2000, 2006, 2013 and 2019, and drew LISA agglomeration map (see Fig 6). In 2000, high-high agglomeration areas were mainly distributed in Shaanxi Province, Chongqing City, Guizhou Province, and Sichuan Province in the western region, and Heilongjiang Province and Jilin Province in the northeast region. Low-low agglomeration areas were mainly concentrated in Shandong Province, Beijing, Jiangsu Province, Shanghai, Tianjin, Fujian Province and other regions along the east coast. From 2000 to 2006, the agglomeration trend in the western region gradually weakened, and the North China region in the low agglomeration area gradually moved to the southeast coastal area. From 2006 to 2013, except for Northeast China, Henan Province, Gansu Province and western Xinjiang still in high-high agglomeration areas, the agglomeration trend of the rest of the western regions was further weakened; the northwest and north China regions in the low-low agglomeration areas migrated to the southeast coastal areas, and the overall agglomeration range shrunk. From 2013 to 2019, high-high agglomeration areas were mainly distributed in the northeast, and low-low agglomeration areas were mainly distributed in the southeast coastal areas. In general, as time goes by, only the SDIGE agglomeration in Northeast China is gradually strengthening, while the agglomeration in other regions is weakening.

### 4.2. SDIGE driver screening and model comparison

#### 4.2.1. SDIGE driver screening

This paper selects six characteristic time points in 2000, 2004, 2008, 2011, 2015 and 2019, and uses the ordinary least squares method to carry out regression tests on 11 driving factors in total from two aspects of socio-economic and geographical conditions and screen out the factors with a higher significance level (see Table 3). According to Table 3, in terms of social economy, four factors, including economic development level (*lnPGDP*), population density (*lnPD*), industrial structure (*lnIS*) and fiscal decentralisation (*lnFD*), passed the significance test at least at the 10% level in six years. In terms of geographical conditions, only topographic relief (*lnTR*) has passed the significance test at the level of 1% in six years. Agricultural mechanisation level (*lnAML*), Intensity of energy consumption (*lnECI*), Environmental regulation (*lnER*), Financial development level (*lnFDL*), Provincial border counties (*PBC*) and Elevation (*lnEL*) did not pass the significance test in all six years. At the same time, according to the requirement that the Variance Inflation Factor (VIF) values of the driving factors required by the GWR model are all less than 7.5, the influencing factors with higher VIF values are eliminated (see Table 4). According to Table 4, the highest VIF value of 11 drivers was 5.660 in the six years, which did not exceed 7.5, thus meeting the requirements of the GWR model. Therefore, this paper finally takes the above five variables as the driving factors of SDIGE and incorporates them into the GWR model to explore their local characteristics and spatial heterogeneity.

**Table 3:** The ordinary least squares analysis results of county SDIGE drivers in China.

Variables	2000		2004		2008		2011		2015		2019	
lnPGDP	-0.786***	(0.000)	-0.955***	(0.000)	-1.059***	(0.000)	-0.883***	(0.000)	-0.968***	(0.000)	-0.788***	(0.000)
lnAML	0.029	(0.283)	0.169***	(0.000)	0.419***	(0.000)	0.479***	(0.000)	0.509***	(0.000)	0.607***	(0.000)
lnPD	-0.181***	(0.000)	-0.171***	(0.000)	-0.178***	(0.000)	-0.220***	(0.000)	-0.370***	(0.000)	-0.292***	(0.000)
lnIS	-0.560***	(0.000)	-0.696***	(0.000)	-0.698***	(0.000)	-0.841***	(0.000)	-0.715***	(0.000)	-1.560***	(0.000)
lnECI	-0.167***	(0.000)	-0.208***	(0.000)	-0.139***	(0.001)	-0.018	(0.661)	-0.081*	(0.053)	0.030	(0.596)
lnER	-0.237***	(0.000)	-0.069	(0.342)	-0.102	(0.252)	0.031	(0.760)	-0.197**	(0.022)	0.112	(0.406)
lnFD	-0.199***	(0.000)	-0.125**	(0.024)	-0.117*	(0.077)	-0.402***	(0.000)	-0.379***	(0.000)	-0.548***	(0.000)
lnFDL	0.136***	(0.000)	0.195***	(0.000)	0.107**	(0.015)	0.027	(0.589)	0.023	(0.748)	-0.158*	(0.062)
lnTR	-0.143***	(0.000)	-0.236***	(0.000)	-0.336***	(0.000)	-0.364***	(0.000)	-0.311***	(0.000)	-0.391***	(0.000)
PBC	-0.074**	(0.012)	-0.033	(0.374)	-0.064	(0.245)	-0.116*	(0.051)	-0.132**	(0.029)	0.049	(0.544)
lnEL	0.094***	(0.000)	0.135***	(0.000)	0.177***	(0.000)	0.166***	(0.000)	0.092***	(0.002)	0.038	(0.377)
Cons	-0.583**	(0.012)	-0.052	(0.865)	0.235	(0.524)	1.729***	(0.000)	2.347***	(0.000)	2.682***	(0.000)
R^2^	0.574		0.527		0.400		0.364		0.360		0.415	
Adj R^2^	0.571		0.525		0.396		0.360		0.357		0.412	
AIC	3764.786		4809.200		6360.758		6790.513		6834.593		8023.338	
AICc	3764.968		4809.382		6360.939		6790.694		6834.774		8023.520	
F-Stat	245.864***		203.622***		121.564***		104.455***		102.922***		129.589***	
K(BP)	292.597***		238.221***		85.440***		118.611***		154.790***		449.855***	

Note: When the K (BP) statistic is significant, the statistical significance of the independent variable needs to be judged by the robust P-value; Robust P-value in brackets;

***, ** and * represent the significance levels of 1%, 5% and 10%.

**Table 4:** VIF results of each driver.

Variables	2000	2004	2008	2011	2015	2019
*lnPGDP*	3.645	3.567	3.118	2.862	2.423	2.188
*lnAML*	1.981	1.817	1.633	1.519	1.652	1.528
*lnPD*	4.632	5.253	5.149	5.414	5.099	5.207
*lnIS*	1.827	1.809	1.893	1.765	1.570	1.710
*lnECI*	3.860	3.558	3.009	2.734	2.498	2.282
*lnER*	2.137	2.083	2.157	2.011	2.082	1.913
*lnFD*	2.521	2.949	2.723	3.095	3.062	2.759
*lnFDL*	1.322	1.238	1.187	1.211	1.248	1.184
*lnTR*	5.387	5.495	5.542	5.532	5.660	5.565
*PBC*	1.059	1.051	1.041	1.041	1.042	1.050
*lnEL*	5.071	4.998	4.856	4.835	4.958	4.843

#### 4.2.2. Model comparison of SDIGE

This paper used ArcGIS10.2 software to conduct geographically weighted regression on the five driving factors that passed the significance test and to obtain local regression parameters (see Table 5). By comparing the regression parameters in Table 5 and Table 3, it is found that the minimum values of *R*^*2*^ and *Adj R*^*2*^ are 0.668 and 0.667 after using the GWR model. Compared to the global regression analysis, the six years have been greatly improved and the fitting degree is better. The AICc value in the six years decreased by 511.798, 695.222, 1162.949, 1443.174, 1260.324 and 1653.670 in turn, with a decrease of more than 2, indicating that the GWR model has a better fitting effect.

**Table 5:** GWR model parameters.

parameters	2000	2004	2008	2011	2015	2019
*R* ^*2*^	0.680	0.676	0.673	0.698	0.668	0.750
*Adj R* ^*2*^	0.679	0.675	0.672	0.698	0.667	0.749
*AICc*	3253.170	4114.160	5197.990	5347.520	5574.450	6369.850
*Bandwidth*	0.115	0.115	0.115	0.115	0.115	0.115
*Residual sum of squares*	564.112	864.538	1479.980	1593.430	1783.160	2644.730
*Sigma*	0.529	0.655	0.856	0.889	0.940	1.145

### 4.3. Analysis of spatial and temporal heterogeneity of SDIGE drivers

#### 4.3.1. Time evolution of each driver

**Fig 7:**
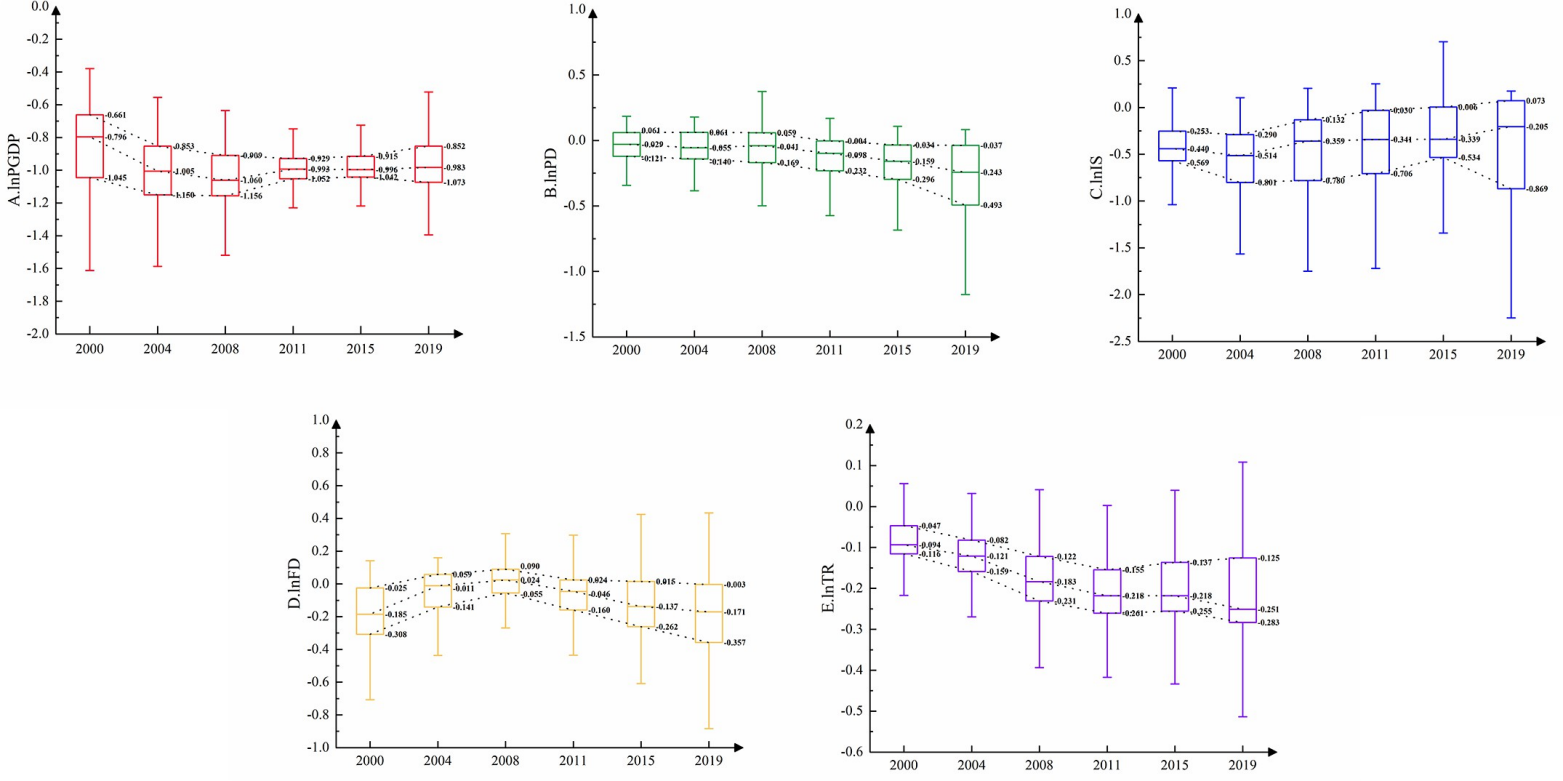
This figure, created using origin software, shows the changing trends of the coefficients of the driving factors of SDIGE in China. The influence coefficients of each variable on SDIGE are plotted on the vertical axis, while the years are plotted on the horizontal axis. The red color represents the influence coefficient and its time variation of lnPGDP on SDIGE, the green color represents lnPD, the blue color represents lnIS, the yellow color represents lnFD, and the purple color represents lnTR.

The GWR model is used to conduct regression analysis on the driving factors of China’s county SDIGE in 2000, 2004, 2008, 2011, 2015 and 2019, to obtain the effect coefficients of each driving factor on SDIGE at different space-time locations. Then a box chart of each coefficient changing with time to analyse its evolution trend is drawn (see Fig 7).

1. The impact of economic development level (*lnPGDP*) on SDIGE is all negatively correlated during the study period, i.e., the improvement of economic development level will reduce the SDIGE of the county. Over time, the regression coefficient and dispersion degree of most counties show a trend of decreasing first and then increasing. The higher the level of economic development is, the stronger the economic agglomeration will be, which will squeeze the space for grain production, thereby reducing SDIGE.
2. The impact of population density (*lnPD*) on SDIGE in most counties in China is negative. The regression coefficient shows a decreasing trend over time, and the degree of dispersion is gradually expanding. This shows that the increase of population density will promote economic development, but has a weak impact on food production, which is similar to the research conclusions of Rahman, Saidi [36] and Peterson [37].
3. Similar to population density, the impact of industrial structure (*lnIS*) on SDIGE in most counties in China is negative, but the regression coefficient shows a rising trend over time. This shows that only a few regions have achieved the goal of "industry feeding agriculture back" in the process of industrialisation.
4. The influence of fiscal decentralisation (*lnFD*) on SDIGE in most counties in China is negative, and the regression coefficient shows an inverted "V" trend over time. At the beginning of the study, fiscal decentralisation had a positive promoting effect on local fiscal agricultural expenditure, thereby improving food production capacity. However, after 2008, due to the continuous increase of grain production costs and the relatively low grain market price, the crowding effect of fiscal decentralisation on grain production began to be greater than the incentive effect, thereby reducing the SDIGE in most regions.
5. Similar to fiscal decentralisation, the impact of terrain relief (*lnTR*) on SDIGE in most counties in China is negative, and the regression coefficient shows a positive "V" trend over time. The terrain has the most direct impact on grain production. The flat terrain and concentrated land are conducive to large-scale mechanised operation. Also, grain production is more likely to develop in the direction of scale, specialisation and commercialisation, such as in Sanjiang Plain and Songnen Plain in Northeast China.

#### 4.3.2. Spatial heterogeneity of each driver

**Fig 8:**
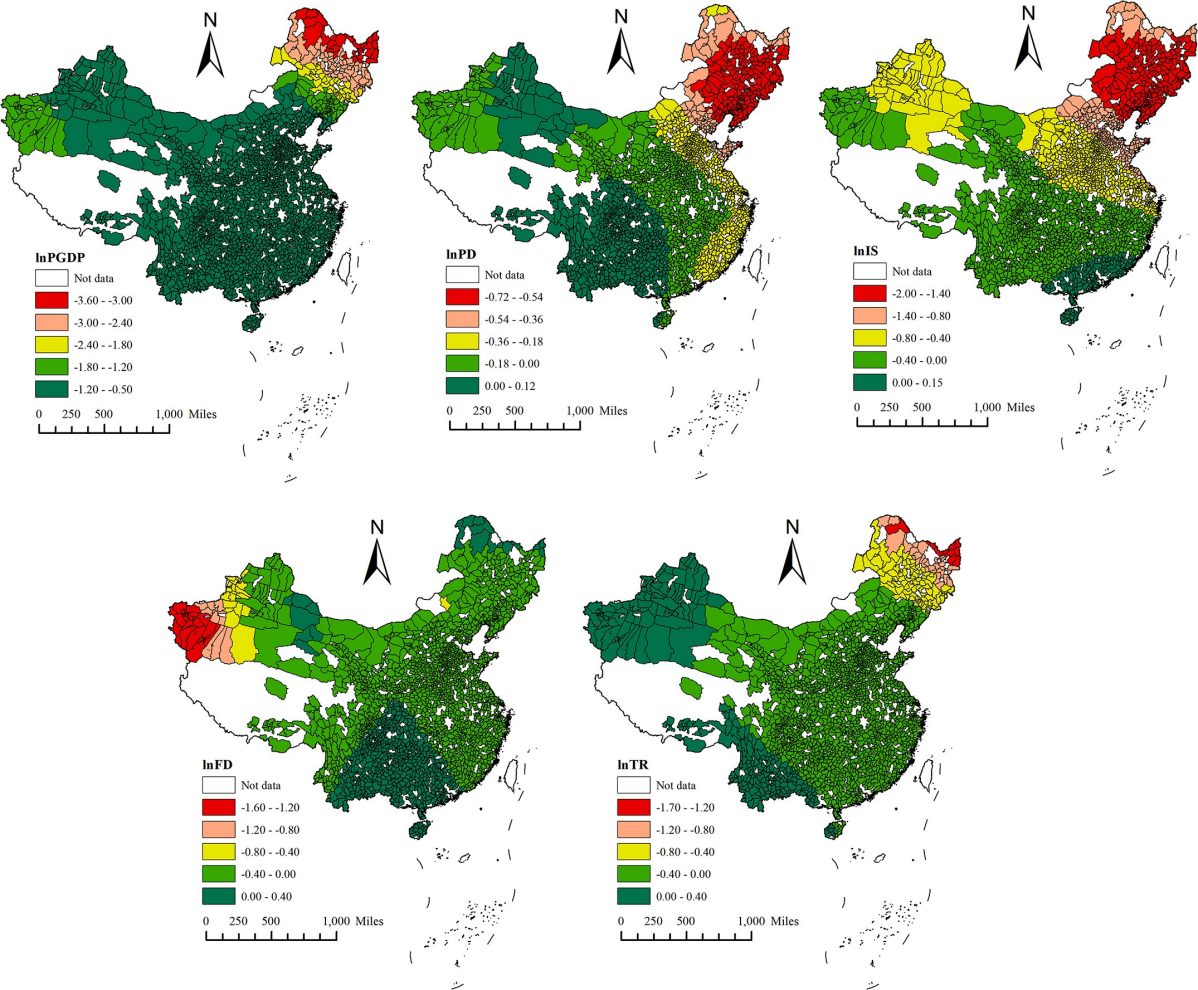
This figure, created using ArcGIS software, illustrates the spatial distribution characteristics of the mean coefficients of lnPGDP, lnPD, lnIS, lnFD, and lnTR on SDIGE. The colors red, pink, yellow, leaf green, and fir green represent increasing mean coefficients.

This paper visualised the average value of the action coefficient of six years through ArcGIS 10.2 to more intuitively explore the difference in the influence degree of each driver of SDIGE in local areas. The temporal and spatial distribution of the mean value of the action coefficient of each driving factor of SDIGE is shown in Fig 8. The counties with a greater impact on SDIGE from the level of economic development (*lnPGDP*) are mainly distributed in northeast China, and include Huma County, Jiayin County, Mohe City, and Tahe County. The regression coefficient shows a consistent negative correlation, with a span of -3.60–-0.50. The counties with large negative impact of population density (*lnPD*) on SDIGE are mainly distributed in northeast China, including Ji’an City, Liuhe County, and Tonghua County. The positive impact is mainly distributed in the southwest and part of the northwest, including Huidong County, Huize County, and Qiaojia County. The spatial distribution characteristics of regression coefficients are obvious. From negative to positive, the regression coefficients are in the order of northeast, east, central and western regions. However, the span of regression coefficients is small, and there are positive and negative distributions between -0.72 and 0.12. The counties where the industrial structure (*lnIS*) has a large negative impact on SDIGE are mainly distributed in the northeast and north China, while the positive impact is mainly distributed in the southeast coastal areas, presenting a spatial pattern of "northeast—southwest." The regression coefficient has a large span, with both positive and negative distributions between -2.00 and 0.15. The counties with greater negative impact of fiscal decentralisation (*lnFD*) on SDIGE are mainly distributed in western Xinjiang and other regions, while the positive impact is mainly distributed in southwest China. The regression coefficient has a small span, with both positive and negative distributions between -1.60 and 0.40. The counties with large negative impact of topographic relief (lnTR) on SDIGE are mainly distributed in parts of the northeast, while the positive impact is mainly distributed in the southwest and parts of the northwest. The regression coefficient has a small span and is distributed between -1.70 and 0.40.

## 5. Discussion

Studying the spatial relationship between grain production and economic development in county-level units in China can assist in the coordinated development of economic growth and grain production. This study utilizes a sample of 2018 county-level units from 31 provinces in mainland China (excluding Hong Kong, Macau, and Taiwan) to examine the spatiotemporal evolution characteristics of the SDIGE in China, as well as its driving factors. The findings of this study can contribute to research support for enhancing food security and promoting sustainable economic development in China or other developing countries.

Firstly, the relationship between grain production and economic development has become a focus of research among scholars. The phenomenon of "economic displacement of grain" has been verified in many studies [38, 39]. Secondly, while rapid industrialization and urbanization can lead to rapid economic development, they can also encroach upon grain production space, resulting in an imbalance between regional economic development and grain production. This viewpoint is similar to the findings of Lu, Liang [40] research.

In terms of the driving forces behind SDIGE, this study found that fiscal decentralization harms the majority of county-level SDIGE indices in China. This is mainly because the increase in fiscal decentralization levels leads to an expansion of fiscal resources for local governments. In the context of political competition, local government officials often prioritize economic growth as a key performance indicator. To achieve this goal, they tend to encroach upon grain production space. This finding is similar to the research conducted by He, Wei [41]. Of course, this study has provided some new findings on the relationship between economic development and grain production. Unlike previous research, the innovative discovery of this study is that in regions with a more developed grain industry (such as China’s northeastern region), the negative impact of economic development level, population density, and industrial structure on SDIGE becomes more pronounced. Overall, this study contributes to providing a certain reference basis for the central government to formulate rational regional development policies or measures, and to orient policy implementation towards the goal of "coordinated development of economy and grain production".

In addition, it is important to acknowledge the limitations of this study, which can inspire further research. Due to data constraints, this study only uses two indicators, economic geographic concentration and grain geographic concentration, to calculate SDIGE, resulting in a relatively simplistic calculation method. Furthermore, the analysis of factors influencing SDIGE in China, especially economic development level, population density, industrial structure, fiscal decentralization, and topographical variation, remains incomplete and requires further refinement. Finally, although this study did not explore this aspect further due to data limitations, it is reasonable to expect that more interesting conclusions will be discovered, which will also have richer practical implications.

## 6. Conclusions and policy implications

### 6.1 Conclusions

This study is based on panel data covering 2018 county-level units in China from 2000 to 2019. It employs methods such as Theil index three-stage nested decomposition and GWR to examine the spatiotemporal evolution characteristics and driving factors of the SDIGE in Chinese counties. This research holds significant importance for comprehensively understanding China’s current food security situation, grasping the coordinated development of grain production and regional economy, and formulating differentiated food policies. The research findings are as follows: (1) In terms of the size of SDIGE, China’s SDIGE shows a development trend of "up—down—up," with the highest SDIGE in Northeast China and the lowest in East China. (2) In terms of spatial distribution, the spatial pattern of "high in the northeast—low in the east coast" of SDIGE in China became increasingly prominent from 2000 to 2019. The spatial pattern of "high in the southwest—low in the northwest" has gradually evolved into "high in the north—low in the northwest." The distribution centre has gradually moved to the northeast, showing a trend of spatial divergence from the northeast to the southwest and centripetal concentration from the northwest to the southeast. (3) In terms of the dynamic evolution of distribution, the SDIGE difference in China from 2000 to 2019 showed the evolution characteristics of first expanding and then shrinking, and the trend of monopolarisation was relatively stable. The dynamic evolution process of the SDIGE time series in the four zones is different due to the superposition and symbiosis of regional space-time characteristics. (4) In terms of spatial difference, the overall difference of SDIGE in China from 2000 to 2019 showed a rising development trend. The average contribution rate of the regional difference to the overall difference was the lowest, maintained at about 17.82%. The average contribution rate of intra-city and inter-county differences to the overall difference is the highest, at about 34.20%. (5) In terms of spatial correlation, Moran’s index of SDIGE in China was significantly positive from 2000 to 2019, and over time, only the SDIGE agglomeration in Northeast China has gradually strengthened, while the agglomeration in other regions has weakened. (6) Each driving factor has obvious spatial heterogeneity characteristics. The level of economic development harms SDIGE, while population density, industrial structure, fiscal decentralisation and topographic relief have positive and negative dual impacts on SDIGE. (7) In terms of spatial characteristics of driving forces, 2018 county units can be divided into five positive and negative influence areas. In terms of negative impact, the spatial characteristics of driving forces can be divided into the northeast region dominated by economic development level, population density, industrial structure and topographic relief, and the western Xinjiang region dominated by fiscal decentralisation. In terms of positive impact, it can be divided into southeast coastal areas dominated by industrial structure, southwest areas dominated by fiscal decentralisation, southwest areas dominated by population density and topographic relief, and the northwest areas.

### 6.2. Policy implications

Based on the spatial distribution characteristics of SDIGE in China and the differential impacts of its driving factors, it is necessary to further strengthen policies to support grain producers, alleviate the imbalance between grain production and economic development, and ensure food security. Firstly, in regions with favourable natural conditions and terrain, such as the central, eastern, and northeastern regions, policies should be optimized to promote urban-rural integration and industrial-agricultural integration, while maintaining a balanced relationship between economic development and grain production. Secondly, from a holistic perspective, it is essential to increase financial support policies and funding for agriculture, and to enhance agricultural production and rural infrastructure development, thereby effectively improving the stability of grain production.

## Supporting information

S1 Data (XLSX)
